# Epidemiology of multiple myeloma in 17 Latin American countries: an update

**DOI:** 10.1002/cam4.1347

**Published:** 2018-03-24

**Authors:** Maria Paula Curado, Max M. Oliveira, Diego R. M. Silva, Dyego L. B. Souza

**Affiliations:** ^1^ Epidemiology and Statistics Group Research Center A.C Camargo Cancer Center São Paulo Brazil; ^2^ International Prevention Research Institute Ecully France; ^3^ National Institute for Science and Technology in Oncogenomics and Therapeutic Innovation Brazil; ^4^ Graduate Program in Public Health School of Public Health University of São Paulo São Paulo Brazil; ^5^ Collective Health Department Federal University of Rio Grande do Norte Natal Brazil

**Keywords:** incidence, mortality, multiple myeloma, trend

## Abstract

The objective of this study was to describe incidence, mortality rates, and trends for multiple myeloma (MM) in Latin America (LA), contributing to better knowledge on the epidemiology of MM in this continent. Incidence data were extracted from the International Agency for Research on Cancer (IARC), for the period 1990–2007. Mortality data were obtained for 17 countries from the World Health Organization, for the period 1995–2013. Annual average percentage change (AAPC) and 95% confidence interval (95% CI) were calculated for incidence and mortality. The average incidence rate of MM was higher in Cali (Colombia). For the age‐group over 60 years old, rates were 14.2 and 12.8 per 100,000 inhabitants for men and women, respectively. Increasing incidence trends were verified for Cali (Colombia). Mortality rates were higher among men; most countries presented increasing trends, and the highest increments were observed in Guatemala (12.5% [95% CI: 10.6; 14.5] in men; 8.8% [95% CI: 7.8; 9.8] in women), Ecuador (5.5% [95% CI: 5.0; 6.0] in men; 3.7 [95% CI: 3.1; 4.3] in women), Paraguay (2.9% [95% CI: 2.3; 3.5] in men; 3.2% [95% CI: 2.1; 4.3] in women), and Brazil (1.4% [95% CI: 1.3; 1.5] in men; 0.9% [95% CI: 0.8; 1.0] in women). Multiple myeloma presented heterogeneous incidence patterns in Cali (Colombia), Quito (Ecuador), and Costa Rica. Increasing mortality trends were verified for most Latin American countries and could be related to limited access to diagnosis and new therapies.

## Introduction

Multiple myeloma (MM) represents, approximately, 1% of all cancers in the world; although rare, it is the second most frequent hematologic neoplasm 1, 2, 3. Incidence is higher in individuals over 60 years old, in men, in the Black race, and in individuals with family history of this malignancy 4, 5, 6, 7, 8.

In the world, in 2012, 144,251 new MM cases were estimated for both sexes, with standardized incidence rates of 1.5/100,000 and 80,019 deaths, with the global standardized mortality rate being 1.0/100,000 1. Incidence rates for White North Americans and for most European countries are similar 1, 2, 3. In South America, the estimated rates are 1.7 for incidence and 1.3/100,000 for mortality 1, 3.

Multiple myeloma incidence has increased in Great Britain, the United States, and in West Europe; this increase was attributed to better accessibility to health services and better MM diagnosis 8, 9, 10. Despite the increasing incidence rates for MM, studies that use population data are more frequent in developed countries 8, 9, 10, 11 than in developing countries 12. Latin America is a geographic area with scarce studies on multiple myeloma, a rare malignancy. Within Latin America, the life expectancy of the population is increasing, and therefore, it is relevant to describe the epidemiological profile of MM in Latin American countries. The aim of this study was to describe incidence, mortality rates, and trends for multiple myeloma in selected countries of Latin America, based on data from the existing Population‐Based Cancer Registries and from the mortality database available at the WHO Web site.

## Methods

An ecological study is presented herein, based on temporal series, which utilized data on multiple myeloma incidence and mortality (C90) 13, 14 from the databases of the International Agency for Research of Cancer (IARC) and World Health Organization (WHO) 15, 16.

Incident cases of MM over a period of 17 years (1990–2007) were extracted from Cancer Incidence in Five Continents—CI5 PLUS 15, which included three PBCRS: two regional registries, Cali (Colombia) and Quito (Ecuador), and one national registry, Costa Rica 15. Regarding mortality, death records of 17 Latin American countries were selected, which represented approximately 90% of the population of Latin America, between 1995 and 2013 (WHO Cancer Mortality Database) 16.

The number of cases was extracted, and age‐adjusted specific rates were calculated for two age‐groups (40–59 and 60+) and to all ages. The age‐adjusted specific rates were calculated using the world standard population, according to sex and for selected geographic areas with available data. Standardized incidence and mortality rate ratios were calculated per sex (male:female) with a 95% confidence interval (95% CI). The annual average percentage change (AAPC) was estimated for mortality and incidence with 95% CI, except for Belize, El Salvador, and Suriname, due to lack of cases in the historical series. Statistical analyses were carried out using the R package epitools version 0.5‐9 17 and the Joinpoint Regression Program software, version 4.5.0.0 18.

## Results

**Figure 1:**
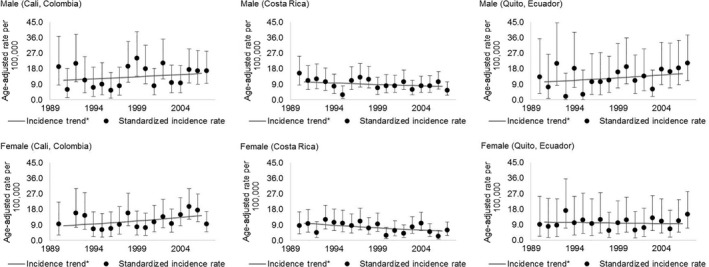
Multiple myeloma age‐adjusted incidence rates (95% confidence interval) for sex, age above 60 years, for Cali (Colombia), Costa Rica, and Quito (Ecuador), for the period 1990–2007. 95% CI: 95% confidence interval. The gray line represents trends over the period.

Between 1990 and 2007, the highest incidence rates of multiple myeloma were observed in Cali (Colombia) and Quito (Ecuador) in the age‐group over 60 years old, with rates ranging from 14.2/100,000 for men to 12.8/100,000 for women. Incidence rate ratios were higher in Quito (Ecuador), 1.4 (95% CI: 1.2; 1.7), and more frequent in men (Table 1, Fig. 1).

**Table 1:** Age standardized incidence rate (ASIR), number of cases (*N*), average annual percent change (AAPC), and incidence rate ratio (SIR) for multiple myeloma, according to age and sex, in Cali (Colombia), Costa Rica, and Quito (Ecuador), for the period 1990–2007

PBCR	Age‐group (years)	Male		Female		SIR (95% CI^a^)
		ASIR (*N*)	AAPC (95% CI^a^)	ASIR (*N*)	AAPC (95% CI^a^)	
Cali (Colombia)	40–59	4.4 (117)	**2.0 (1.4; 2.6)**	3.1 (97)	**1.6 (0.3; 2.9)**	**1.4 (1.1; 1.8)**
	60+	14.2 (170)	**2.0 (0.9; 3.1)**	12.8 (186)	**3.5 (2.7; 4.3)**	1.1 (0.9; 1.4)
	Total	2.6 (306)	**2.0 (1.1; 2.8)**	2.0 (293)	**2.8 (2.0; 3.6)**	**1.3 (1.1; 1.5)**
Costa Rica	40–59	2.1 (115)	**−3.6 (−4.6; −2.5)**	1.6 (90)	0.8 (−0.4; 2.0)	**1.3 (1.1; 1.6)**
	60+	9.1 (237)	**−2.0 (−2.6; −1.4)**	7.3 (202)	**−3.9 (−4.6; −3.2)**	**1.2 (1.1; 1.4)**
	Total	1.5 (366)	**−2.7 (−3.1; −2.4)**	1.2 (303)	**−2.5 (−3.1; −1.9)**	**1.3 (1.1; 1.4)**
Quito (Ecuador)	40–59	3.6 (65)	**8.5 (7.5; 9.6)**	2.3 (46)	**−1.1 (−2.1; −0.1)**	**1.6 (1.2; 2.1)**
	60+	13.5 (118)	**2.6 (1.7; 3.4)**	10.3 (109)	**−0.7 (−1.1; −0.2)**	**1.3 (1.1; 1.6)**
	Total	2.3 (194)	**4.1 (3.5; 4.7)**	1.6 (159)	**−0.9 (−1.5; −0.3)**	**1.4 (1.2; 1.7)**

a 95% confidence interval. Bold represents statistically significant values (*p*<0.05).

**Figure 2:**
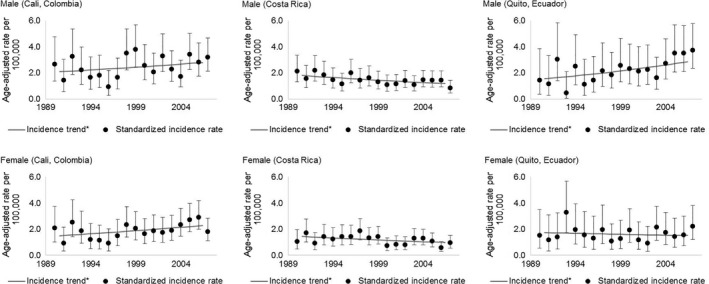
Multiple myeloma age‐adjusted incidence rates (95% confidence interval), by sex, for Cali (Colombia), Costa Rica, and Quito (Ecuador), for the period 1990–2007. 95% CI: 95% confidence interval. The gray line represents trends over the period.

Increasing incidence trends for MM were observed, for both sexes, in Cali (2.0% [95% CI: 1.1; 2.8] in men; 2.8% [95% CI: 2.0; 3.6] in women), higher in women. Incidence trends by age‐group followed similar patterns in Cali (Colombia), Costa Rica, and Quito (Ecuador) (Table 1, Figs. 1 and 2).

Between 1995 and 2013, the highest MM mortality rates were observed in Chile (15.1/100,000 in men and 11.9/100,000 in women). Mortality rate ratios were higher in men for all countries and statistically significant (higher than 1), for most countries studied (Table 2).

**Table 2:** Age standardized mortality rate (ASMR) per 100,000, number (*N*) of deaths and mortality rate ratio (SMR) for multiple myeloma, by sex and age‐group, for 17 Latin American populations, in the period 1995–2013

Population	Data availability	Age‐groups	ASMR (*N*)		SRM (95% CI*)
			Male	Female	
Argentina	1997–2013	40–59	1.5 (1028)	1.1 (805)	**1.4 (1.2; 1.5)**
		60+	8.9 (3791)	6.5 (4068)	**1.4 (1.3; 1.4)**
		Total	1.3 (4896)	1.0 (4910)	**2.2 (2.1; 2.3)**
Belize	1997–2013	40–59	1.2 (4)	1.4 (4)	0.9 (0.2; 3.2)
		60+	3.7 (5)	2.9 (4)	1.3 (0.3; 4.9)
		Total	0.7 (9)	0.6 (8)	1.2 (0.6; 2.4)
Brazil	1996–2013	40–59	1.5 (4680)	1.1 (3907)	**1.4 (1.3; 1.4)**
		60+	8.4 (10977)	6.6 (11655)	**1.3 (1.2; 1.3)**
		Total	1.2 (16018)	1.0 (15819)	**1.2 (1.2; 1.2)**
Chile	1997–2013	40–59	2.3 (724)	1.6 (517)	1.0 (0.9; 1.1)
		60+	15.1 (2383)	11.9 (2584)	1.0 (1.0; 1.1)
		Total	2.2 (3134)	1.6 (3120)	1.0 (1.0; 1.1)
Colombia	1997–2013	40–59	1.3 (880)	1.0 (731)	**1.3 (1.2; 1.4)**
		60+	7.4 (2011)	5.8 (1972)	**1.3 (1.2; 1.4)**
		Total	1.1 (2954)	0.9 (2752)	**1.2 (1.2; 1.3)**
Costa Rica	1997–2013	40–59	1.6 (117)	1.3 (99)	1.2 (1.0; 1.6)
		60+	12.4 (444)	8.8 (355)	**1.4 (1.2; 1.6)**
		Total	1.7 (571)	1.2 (457)	**1.4 (1.3; 1.6)**
Ecuador	1997–2013	40–59	0.9 (168)	0.6 (120)	**1.5 (1.2; 1.9)**
		60+	4.4 (415)	3.4 (362)	**1.3 (1.1; 1.5)**
		Total	0.7 (600)	0.5 (500)	**1.4 (1.3; 1.6)**
El Salvador	1997–2013	40–59	0.2 (16)	0.2 (18)	1.0 (0.5; 2.0)
		60+	0.8 (35)	0.5 (28)	1.6 (1.0; 2.7)
		Total	0.1 (53)	0.1 (49)	1.0 (0.7; 1.5)
Guatemala	2000–2013	40–59	0.4 (40)	0.2 (31)	**2.0 (1.2; 3.2)**
		60+	1.2 (68)	1.1 (70)	1.1 (0.8; 1.5)
		Total	0.2 (111)	0.2 (113)	1.0 (0.8; 1.2)
Mexico	1998–2013	40–59	1.4 (2047)	1.2 (1789)	**1.2 (1.1; 1.2)**
		60+	6.5 (4308)	4.8 (3782)	**1.4 (1.3; 1.4)**
		Total	1.0 (6559)	0.8 (5702)	**1.3 (1.2; 1.3)**
Nicaragua	1997–2013	40–59	0.6 (37)	0.4 (30)	1.5 (0.9; 2.4)
		60+	2.4 (60)	1.3 (41)	**1.9 (1.2; 2.8)**
		Total	0.4 (105)	0.2 (74)	**2.0 (1.6; 2.6)**
Panama	1998–2013	40–59	1.8 (88)	1.2 (61)	**1.5 (1.1; 2.1)**
		60+	10.2 (260)	10.2 (210)	1.0 (0.8; 1.2)
		Total	1.5 (356)	1.1 (275)	**1.4 (1.2; 1.6)**
Paraguay	1996–2013	40–59	0.7 (62)	0.6 (48)	1.2 (0.8; 1.7)
		60+	3.9 (144)	3.6 (149)	1.1 (0.9; 1.4)
		Total	0.6 (215)	0.5 (200)	1.2 (1.0; 1.4)
Peru	1999–2013	40–59	0.9 (335)	0.6 (228)	**1.5 (1.3; 1.8)**
		60+	6.2 (1086)	3.6 (733)	**1.7 (1.6; 1.9)**
		Total	0.9 (1468)	0.5 (990)	**1.8 (1.7; 1.9)**
Suriname	1995–2013	40–59	1.4 (12)	1.0 (9)	1.4 (0.6; 3.3)
		60+	10.7 (41)	2.8 (14)	**3.8 (2.0; 7.2)**
		Total	1.5 (53)	0.5 (24)	**3.0 (1.9; 4.8)**
Uruguay	1997–2010	40–59	2.1 (106)	1.8 (104)	1.2 (0.9; 1.5)
		60+	14.2 (535)	10.5 (641)	**1.4 (1.2; 1.5)**
		Total	2.0 (648)	1.6 (747)	**1.3 (1.1; 1.4)**
Venezuela	1996–2013	40–59	1.6 (652)	1.3 (571)	**1.2 (1.1; 1.4)**
		60+	8.3 (1386)	6.8 (1371)	**1.2 (1.1; 1.3)**
		Total	1.3 (2109)	1.0 (1988)	**1.3 (1.2; 1.4)**

95% confidence interval.

**Figure 3:**
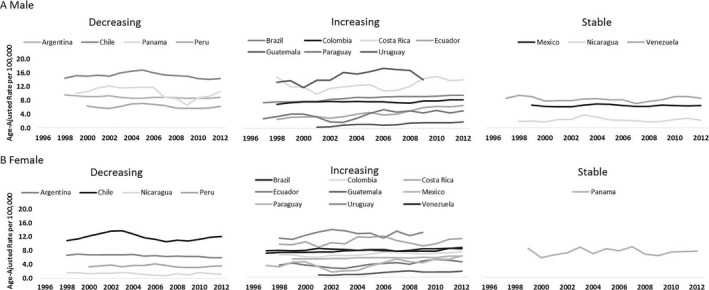
Temporal trends in multiple myeloma mortality, according to sex, and age above 60 years, in 17 Latin American countries, for the period 1995–2013. (A) Male; (B) Female.

Of the 17 studied countries, there was no clear pattern in mortality trends (Fig. 3). For the age‐group over 60 years old, increases were observed in men of seven countries (Brazil, Colombia, Costa Rica, Ecuador, Guatemala, Paraguay, and Uruguay), and decreases were observed in four countries (Argentina, Chile, Panama, and Peru). In women, eight countries presented increasing trends (Brazil, Colombia, Costa Rica, Ecuador, Guatemala, Mexico, Paraguay, and Uruguay), while four countries (Argentina, Chile, Nicaragua, and Peru) presented decreasing trends. The highest decline was observed for men, in Panama (−2.2%; 95% CI:−2.8;−1.5), while the highest increase was verified in Guatemala (15.1% [95% CI: 13.1; 17.2] in men; 10.2% [95% CI: 8.9; 11.4] in women) (Table 3).

**Table 3:** Multiple myeloma mortality trends, by sex and age‐group, for 17 Latin American populations, in the period 1995–2013

Population	Data availability	Age‐groups (years)	AAPC (95% CI)	
			Male	Female
Argentina	1997–2013	40–59	**−0.8 (−1.0; −0.6)**	**−2.0 (−2.1; −1.8)**
		60+	**−0.6 (−0.7; −0.5)**	**−1.0 (−1.1; −0.9)**
		Total	**−0.6 (−0.7; −0.5)**	**−1.1 (−1.2; −1.0)**
Brazil	1996–2013	40–59	**0.4 (0.3; 0.6)**	0.2 (−0.0; 0.5)
		60+	**1.8 (1.7; 1.9)**	**1.2 (1.1; 1.3)**
		Total	**1.4 (1.3; 1.5)**	**0.9 (0.8; 1.0)**
Chile	1997–2013	40–59	**−0.9 (−1.2; −0.5)**	**−1.1 (−1.6; −0.6)**
		60+	**−0.3 (−0.5; −0.1)**	**−0.5 (−0.8; −0.1)**
		Total	**−0.4 (−0.7; −0.2)**	**−0.6 (−0.9; −0.3)**
Colombia	1997–2013	40–59	**−0.9 (−1.2; −0.5)**	**−0.6 (−1.0; −0.1)**
		60+	**0.7 (0.5; 0.8)**	**1.3 (1.1; 1.5)**
		Total	**0.3 (0.1; 0.4)**	**0.8 (0.6; 1.0)**
Costa Rica	1997–2013	40–59	**1.9 (0.8; 2.9)**	−0.2 (−0.8; 0.4)
		60+	**1.0 (0.5; 1.5)**	**0.8 (0.4; 1.2)**
		Total	**1.1 (0.5; 1.6)**	**0.6 (0.3; 1.0)**
Ecuador	1997–2013	40–59	**3.1 (2.2; 4.1)**	**5.5 (5.2; 5.9)**
		60+	**6.8 (6.3; 7.2)**	**3.2 (2.5; 3.9)**
		Total	**5.5 (5.0; 6.0)**	**3.7 (3.1; 4.3)**
Guatemala	2000–2013	40–59	**9.2 (7.5; 10.9)**	**9.3 (6.9; 11.8)**
		60+	**15.1 (13.1; 17.2)**	**10.2 (8.9; 11.4)**
		Total	**12.5 (10.6; 14.5)**	**8.8 (7.8; 9.8)**
Mexico	1998–2013	40–59	**0.4 (0.1; 0.6)**	−0.1 (−0.3; 0.2)
		60+	0.0 (−0.1; 0.2)	**1.0 (0.8; 1.1)**
		Total	0.0 (−0.1; 0.2)	**0.5 (0.4; 0.7)**
Nicaragua	1997–2013	40–59	−0.4 (−2.0; 1.2)	**7.2 (6.0; 8.5)**
		60+	0.6 (−0.4; 1.5)	**−2.2 (−3.1; −1.3)**
		Total	−0.1 (−0.9; 0.8)	**1.1 (0.6; 1.6)**
Panama	1998–2013	40–59	**−5.2 (−6.5; −4.0)**	0.2 (−1.3; 1.8)
		60+	**−2.2 (−2.8; −1.5)**	0.3(−0.4; 0.9)
		Total	**−2.6 (−3.2; −2.1)**	0.2 (−0.3; 0.8)
Paraguay	1996–2013	40–59	0.9 (−0.3; 2.2)	−0.4 (−1.9; 1.0)
		60+	**4.0 (2.8; 5.2)**	**4.4 (3.0; 5.9)**
		Total	**2.9 (2.3; 3.5)**	**3.2 (2.1; 4.3)**
Peru	1999–2013	40–59	−0.2 (−0.8; 0.4)	0.1 (−0.5; 0.6)
		60+	**−0.6 (−1.0; −0.1)**	**−0.6 (−1.0; −0.2)**
		Total	−0.4 (−0.8; 0.1)	−0.1 (−0.5; 0.2)
Uruguay	1997–2010	40–59	**−1.1 (−2.0; −0.2)**	**3.6 (2.4; 4.8)**
		60+	**2.3 (1.7; 2.8)**	**0.7 (0.3; 1.1)**
		Total	1.6 (0.9; 2.2)	**1.6 (1.1; 2.0)**
Venezuela	1996–2013	40–59	**1.3 (1.0; 1.7)**	**0.8 (0.4; 1.2)**
		60+	−0.2 (−0.5; 0.1)	**0.2 (0.0; 0.3)**
		Total	0.2 (0.0; 0.4)	**0.3 (0.2; 0.4)**

AAPC, average annual percentage of change.

## Discussion

The incidence of multiple myeloma in Cali (Colombia), Costa Rica, and Quito (Ecuador) occurred more frequently in the age‐group over 60 years of age, with higher rates in men, similar to other studies 9, 10. The known epidemiological characteristics of MM include higher incidence in males and the elderly (≥60 years of age).

Increasing incidence trends were detected in Cali and in Quito for men; decreasing trends were verified in Costa Rica, for both genders, and in Quito, for women.

Risk factors associated with MM include family history of lymphoid malignancy and ethnicity, being more common in the Black race. Other risk factors include occupational and environmental exposure to benzene, pesticides, DDT, petroleum derivative, and ionizing radiation [19, 20, 21, 22, 23]. The accepted risk factors for multiple myeloma are aging, male gender, Black race, and positive family history. Possible associated risk factors are overweight and obesity, low consumption of fish and green vegetables, AIDS, and herpes zoster 22, 23, 24, 25. Consumption of tobacco 26 was inconclusive, while alcohol consumption could be associated with reduced risk 27. An ecological study that analyzed data from 175 countries identified an association between low ultraviolet B and vitamin D and higher incidence of MM 28, which could explain the differences in incidence across countries.

In Latin America, a case–control from Uruguay indicated elevated risk of MM in those who consumed more processed meat, red meat, and milk—the pattern of risk food was driven by red meat 29. The different prevalence of these risk factors could partially explain the differences observed in LA countries 30, 31.

This heterogeneous pattern of MM incidence and mortality could reflect limited access to diagnosis and treatment, and maybe some incompleteness of the PBCRs and in mortality databases. Increased incidence in European countries, in the United States, and in China indicates that access to health services leads to more precise diagnosis and early treatment, which could explain the increase in incidence 9, 10, 32.

Another hypothesis for the differences between incidence and mortality is racial composition. An American study demonstrated increased MM incidence, which is higher in non‐Hispanic White individuals, for both sexes, and in Black men 10. Black patients in America were found to be 37% less likely to undergo stem cell transplantation and 21% less likely to be treated with bortezomib and lenalidomide 33, 34, and therefore, mortality rates are higher in people of the Black race.

The highest MM mortality rates were observed in men over the age of 60, increasing with age, similar to incidence. However, heterogeneity in mortality and incidence rates suggests gender differences could be due to delays in access to diagnosis and treatment 35, 36. A Brazilian study showed the effectiveness of reference centers for patients with multiple myeloma, with reduced waiting times until bone marrow transplantation 37. The increase in incident rates over the age of 60 is related to increased life expectancies. 38, 39.

Changes in MM treatment have recently affected mortality. Studies have shown an increase in survival rates, when stratifying by periods according to the available treatments 40, 41, 42, 43. However, stratification by age and ethnic group revealed that only patients under the age of 65 and non‐Hispanic White individuals presented significantly better survival 10. Also, the introduction of new medications, for example bortezomib, favored the increase in survival in intermediate‐ or high‐risk myeloma cases 41, 42, 43, 44, 45, although new medications are expensive and not affordable to all patients.

Access to new drugs and differences in regulations across Latin American countries could have also influenced the differences observed in mortality 46.

The increasing incidence and mortality trends in the three cities (Cali, Quito, and Costa Rica) indicate a clear necessity of better organizing access to diagnosis and treatment for this malignancy. In Latin America, fragmented structures are present with consequent unequal allocation of human and material resources in large urban centers 47, 48. Moreover, there are few hematologists in Latin America, with estimates of 0.9 hematologists per 100,000 inhabitants, while the US counts with 2.2/100,000 49. Brazilian, Mexican, and Peruvian studies indicate that delays in pathological evaluations affect considerably diagnosis and treatment 50, reducing survival rates.

Regarding mortality data quality, differences were detected in coverage and completeness in the 17 countries studied herein, varying from 55% completeness in the Dominican Republic to 90% in Argentina, Chile, Costa Rica, Mexico, Uruguay, and Venezuela. Moreover, the percentage of ill‐defined deaths varied from 5% (Costa Rica and Mexico) to 24% (El Salvador) 16. Despite these differences, data were validated by International Organizations 1, 2, 3 and can be used to describe MM mortality in 17 Latin American countries.

An ecological study was presented herein, with scarce data on incidence and more comprehensive data on LA mortality. The existing socioeconomic differences across Latin American countries are reflected in the quality of mortality data 51. For cancer incidence estimates, coverage of LA PBCRs is limited to approximately 20% 46.

Despite these limitations, this study described MM incidence in three cities and MM mortality trends for 17 Latin American countries. Both incidence and mortality presented differences, with increasing incidence trends in two of three cities (except Costa Rica). Increasing MM mortality was verified in seven countries, which could be related to late diagnosis and barriers to treatment and new drugs.

This study described multiple myeloma incidence in three cities and mortality in 17 Latin American countries. MM is a rare neoplasm that is more frequent in age‐groups over 60 years old. The expected increase in Latin American life expectancy will certainly increase the incidence of MM.

## Conflict of Interest

None declared.
